# Lung ultrasound teaching in medical education: a pilot study at a Brazilian medical school

**DOI:** 10.36416/1806-3756/e20230382

**Published:** 2024-05-08

**Authors:** Gabrielle Turnes Pereira Demetrio, Ana Cristina Burigo Grumann, Mariângela Pimentel Pincelli, Leonardo Jonck Staub

**Affiliations:** 1. Universidade Federal de Santa Catarina - UFSC - Florianópolis (SC) Brasil.; 2. Hospital Regional de São José Dr. Homero de Miranda Gomes, São José (SC) Brasil.; 3. Unidade de Terapia Intensiva, Hospital Nereu Ramos, Florianópolis (SC) Brasil.

**Keywords:** Ultrasonography, Lung, Students, medical, education, medical

## Abstract

**Objective:**

To evaluate cognitive learning, ability to perform and interpret lung ultrasound exams, and self-perception of learning among medical students after a short pedagogical intervention at a medical school in Brazil.

**Methods:**

An experimental pilot study was conducted with medical students at different stages of their education (basic cycle, clinical cycle, and medical internship). The participants underwent a cognitive test before and after the intervention, a practical test, a test to recognize lung ultrasound pathologies, and a qualitative evaluation test at the end of the intervention. Statistical analysis was performed using a significance level of p < 0.05.

**Results:**

A total of 42 students were included in the study, with a median age of 23 years and a predominance of males. The mean score of the pre-intervention cognitive test was 2.97 ± 0.87, and that of the post-intervention test was 6.57 ± 1.41, showing significant improvement (p < 0.001). The score of the practical test and that of the recognition of pathologies test also showed significant improvement after the intervention. There was no significant difference in execution time between the groups. Students in the clinical cycle had a better self-perception of learning.

**Conclusions:**

Theoretical teaching and practical training of lung ultrasound in a short pedagogical intervention can improve cognitive performance, practical skills, and interpretation of the exam. The level of learning achievement was higher among more advanced students in medical education. Additionally, the students in the clinical cycle had a better perception of their learning.

## INTRODUCTION

Point-of-care ultrasound (POCUS) is a tool used by non-imaging specialist physicians to answer clinical questions and aid in medical decision-making. It enhances the accuracy of bedside diagnoses, enables real-time monitoring of critically ill patients, and improves the safety of guided procedures.^1^

Initially established in emergency medicine with the Focused Assessment with Sonography for Trauma (designated FAST) protocol,^2^ POCUS has now become an essential component of clinical evaluation and critical patient assessment in ERs and ICUs.^3^ ^-^ ^5^

As a consequence of the inclusion of POCUS in daily clinical practice, medical education programs have also started incorporating POCUS training into their curricula, especially in the United States.

Even though we do not have a Brazilian consensus on the inclusion of POCUS in medical undergraduate education, with the increasing number of publications on the subject it is becoming clear that integrating POCUS education into medical undergraduate programs can enhance the performance of and understanding of physical examinations by medical students, along with a subjective improvement in their confidence in examinations.^7^

Various models for incorporating ultrasound into the curriculum have been proposed in the literature, with a focus on longitudinal integration from basic disciplines such as anatomy to clinical practice.^6^ ^,^ ^8^ Despite the emphasis on the longitudinal model, brief training sessions covering cognitive, practical, and clinical integration aspects are already capable of positively impacting diagnostic accuracy of students and assisting in decision-making. This highlights the need to encourage the inclusion of training, even if brief, in undergraduate curricula.^9^

Among the numerous challenges in implementing POCUS education in Brazil, the lack of official POCUS training or certification, the limited number of qualified professionals to teach the subject, and the absence of a consensus on basic competencies for some specialties are limiting factors for its widespread use.^9^

The main objective of this study was to evaluate the learning of lung ultrasound and the bedside lung ultrasound in emergency (BLUE) protocol^12^ among medical students at different stages of their education at the Federal University of Santa Catarina, located in the city of Florianópolis, Brazil. The secondary objectives were to compare the learning capacity in terms of cognitive aspects, practical skills, and the ability to recognize lung ultrasound pathologies among students at different stages of their medical education. Additionally, the study aimed to evaluate the students’ perception of the teaching and the learning methods employed.

## METHODS

An experimental pilot study was conducted at the Federal University of Santa Catarina involving medical students at different stages of their education. The study received ethical approval of the Research Ethics Committee of the Federal University of Santa Catarina (CAE n. 96912918.7.0000.0121), and informed consent was obtained from the participants.

The study included medical students at the 4th, 5th, 7th, 10th, and 11th semesters with the objective of approaching a sample of the basic cycle (4th and 5th semesters; Group A), the clinical cycle (7th semester; Group B), and the medical internship cycle (10th and 11th semesters; Group C). Students who had already undergone some practical ultrasound training were excluded.

In total, the study included 57 participants: 21 in Group A, 16 in Group B, and 20 in Group C.

The study consisted of a short pedagogical intervention focused on lung ultrasound and the BLUE protocol, in four meetings. The intervention included reading two articles, one lecture, and one session of hands-on training. The article “Relevance of Lung Ultrasound in the Diagnosis of Acute Respiratory Failure (The BLUE Protocol)”^12^ ^)^ and the article “Lung ultrasound in critically ill patients: a new diagnostic tool”^13^ were shared with the students. The lecture provided an overview of lung ultrasound principles, indications, techniques, and interpretation of findings, lasting two hours. The hands-on training session lasted 90 minutes, during which students, in groups of 8 to 10, had the opportunity to perform and interpret lung ultrasound exams under the guidance of experienced instructors.

Before and after the intervention, the participants underwent several assessments to evaluate their learning outcomes. These assessments included a cognitive test (Test 1), a practical test (Test 2), a test to recognize lung ultrasound pathologies (Test 3), and a qualitative evaluation test (Test 4).

**Figure 1:**
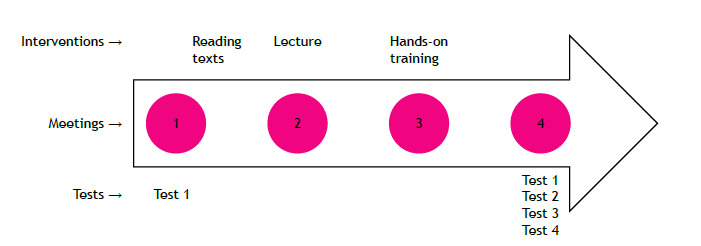
Study design showing the timeline of the study with chronological orientation of the four meetings and moments of application of Tests 1, 2, 3 and 4.

The cognitive test consisted of 22 questions that assessed the participants’ theoretical knowledge of lung ultrasound and the BLUE protocol. Test 1 was performed before (Score 1) and after (Score 2) the intervention. The practical test (Test 2; Score 3) assessed the participants’ ability to perform the BLUE protocol on a standardized patient (healthy voluntary live model). Participants were evaluated on their technique, accuracy, and interpretation of findings. The test to recognize lung ultrasound pathologies (Test 3; Score 4) presented to the participants five images of lung ultrasound findings and asked them to identify the corresponding pathology. The qualitative evaluation test (Test 4) aimed to gather feedback from the participants regarding the teaching methods, content, and overall learning experience. It included a qualitative questionnaire using a Likert scale about the perspective of learning (Figure 1).

Statistical analyses were conducted using the IBM SPSS Statistics software package, version 2019 (IBM Corporation, Armonk, NY, USA). To assess the normality of variables, a Kolmogorov-Smirnov test was employed. Descriptive analyses were performed for both categorical and numerical variables. Categorical variables were presented as absolute numbers and frequencies, while numerical variables were reported in terms of means and standard deviations for those with a normal distribution. For numerical variables exhibiting non-normal distribution, medians and interquartile ranges were utilized. To compare Score 1 and Score 2 across the three groups (A, B, C) for Test 1, execution time, and Test 3 variables, the chi-square test was employed for categorical variables. Meanwhile, Student’s t-test or ANOVA with Bonferroni correction was used for the comparison of numerical variables in repetitive measures. For Score 3 and Score 4, which displayed non-normal distribution, the Mann-Whitney and Kruskal-Wallis tests were applied for comparisons between two or more groups, respectively.

The sample size calculation was based on a gain of at least 2 points between the initial and final scores, considering that the initial score of 5 is a standard deviation of 2, with a power of 80% and a bilateral confidence interval of 95%, which indicated that each group should have 16 members. The level of statistical significance was considered at p < 0.05.

## RESULTS

All stages of the study were completed by 73.7% (42 of the initial 57) students: 13 in Group A (61.9%), 14 in Group B (87.5%), and 15 in Group C (75.0)%. The distribution between the different cycles of the medical course was equitable, with a median age of 23 years and a predominance of males, as shown in Table 1.

**Table 1:** Demographic data of participants.

Variable	General	Group A	Group B	Group C
Semester		4 and 5	7	10 and 11
Education level		Basic cycle	Clinical cycle	Medical internship
Participant,n	42	13	14	15
Age, years*	23 (22-24)	21 (20.5-22)	23 (22-25)	24 (23-26)
Sex, male/female, n/n	29/13	9/4	12/2	8/7

*Data presented as median (IQR).

All test results were converted into a score ranging from 0 to 10. Score 1, which represents the total number of correct answers in Test 1 before the pedagogical intervention, had a general mean of 3.83 ± 1.23. The subgroup means for groups A, B, and C were, respectively, 2.97 ± 0.87, 4.19 ± 1.38, and 4.24 ± 1.03. Score 2, representing the total number of correct answers in Test 1 after the pedagogical intervention, had a general mean of 7.22 ± 1.33. The subgroup means for groups A, B, and C were 6.57 ± 1.41, 6.98 ± 1.49, and 8.00 ± 0.59, respectively.

**Figure 2:**
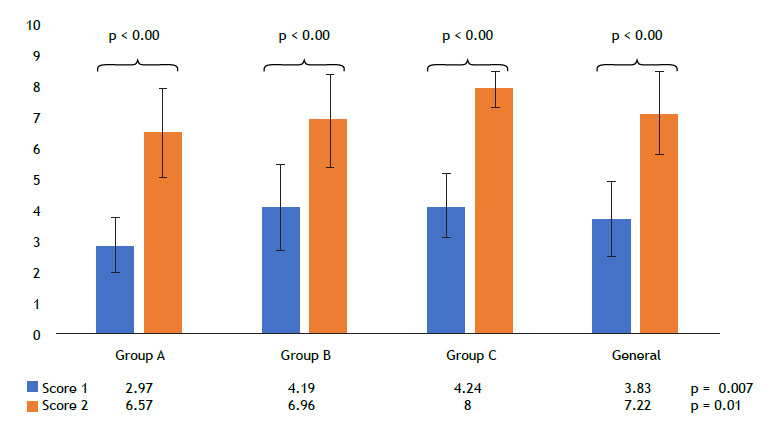
Mean pre- and post-intervention scores in Test 1 (cognitive test) in the sample as a whole and by group.

In the comparative analysis of Score 1 (cognitive assessment) among groups with a normal distribution, ANOVA with Bonferroni correction revealed a significant difference in grades between the groups (p = 0.007). The same was observed for Score 2 (cognitive assessment after intervention; p = 0.01). In the discriminant analysis of variance for Score 1 between each pair of groups, there was a statistical difference between groups A and B (p = 0.02) and between groups A and C (p = 0.01), but not between groups B and C (p = 1.00). The comparison of Score 2 between the three different groups showed a statistical difference only between groups A and C (p = 0.01), but not between groups A and B (p = 1.00) or between groups B and C (p = 0.09), as depicted in Figure 2 and Table 2. The t-test comparing the means of Score 1 and Score 2 showed statistical significance between all of the groups (p < 0.001).

**Table 2:** Comparison scores among all groups, and execution time of the bedside lung ultrasound in emergency protocol.

Score	Group A	p (A and B)	Group B	p (B and C)	Group C	p (A and C)	p (A, B and C)	General
Score 1	2.97 ± 0.87	0.02	4.19 ± 1.38	1.00	4.24 ± 1.03	0.01	0.007	3.83 ± 1.23
Score 2	6.57 ± 1.41	1.00	6.98 ± 1.49	0.09	8.00 ± 0.59	0.01	0.01	7.22 ± 1.33
p (Score 1 and Score 2)	< 0.01		< 0.01		< 0.01			< 0.01
Score 3	8.18 (6.36-10.0)	0.22	9.09 (8.86-10.0)	0.037	10 (9.09-10.0)	0.015	0.013	9.09 (8.18-10.0)
Score 4	6 (4-8)	0.46	6 (4-8)	0.026	8 (8-10)	0.005	0.008	8 (4-8)
Execution time, s	380 ±98		333 ± 70		326 ± 107		0.199	346 ± 94

Score 1: score of the first assessment of cognitive knowledge pre-pedagogical intervention; Score 2: score of the repetition of the cognitive test after the pedagogical intervention; Score 3: score from the practical performance test; and Score 4: score from the recognizing ultrasound patterns test. Data are expressed as mean ± SD or as median (IQR).

For Score 3, which had a non-normal distribution, the Kruskal-Wallis test was used to assess whether there was a difference between the groups. The test revealed a significant difference (p = 0.013), as shown in Table 2. In multiple comparisons with the Mann-Whitney test, Score 3 showed a statistical difference between groups A and C (p = 0.015) and between groups B and C (p = 0.037), but not between groups A and B (p = 0.22).

**Figure 3:**
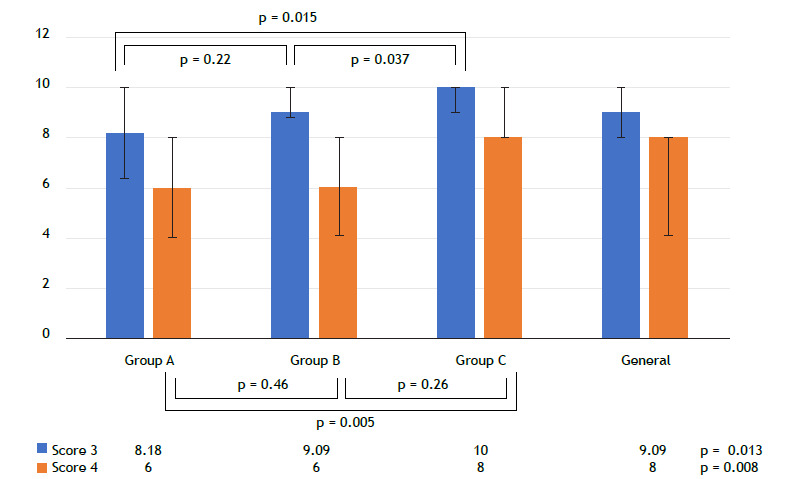
Median of Score 3 and Score 4 in the sample as a whole and by group.; Score 3: score from the practical performance test; and Score 4: score from the recognizing ultrasound patterns test.

Regarding the assessment of the ability to recognize ultrasound patterns evaluated in Score 4, there was a significant difference between the scores of the groups (p = 0.008). When comparing groups pairwise, differences were observed between groups A and C (p = 0.005) and between groups B and C (p = 0.026), but not between groups A and B (p = 0.46). The scores for each group are shown in Table 2 and Figure 3.

The mean execution time of the BLUE protocol in the practical test during the 4th meeting was 346 ± 94 seconds, with means of 380 ± 98 s for group A, 333 ± 70 s for group B, and 326 ± 107 s for group C. Runtime data were missing for 5 participants (1, 2, and 2 in groups A, B, and C, respectively). There was no difference in execution time between the groups (ANOVA; p = 0.199).

In Test 4, when students were asked to report in descending order of importance which method provided them with greater learning, practical training was considered the most effective method for learning by 73.8% (n = 31) of the participants, followed by written material in 59.5% (n = 25) and classes in 54.8% (n = 23).

The subjective assessment of the importance of learning POCUS and the ability to benefit from the course were evaluated in Test 4 using a Likert scale. Thirty-nine participants (92.9%) fully agreed that the course provided relevant knowledge for medical practice that had not yet been addressed in the current curriculum of the medical school. There was almost unanimous agreement that POCUS improves the accuracy of clinical diagnosis (n = 41; 97.6%). Furthermore, there was total agreement that the course content was suitable for learning the BLUE protocol (100%) and that the inclusion of POCUS content in medical education is relevant (100%).

When asked about whether the practical training time was sufficient, 28 (66.6%) of the students partially agreed or fully agreed with the statement, while 7 (16.7%) disagreed. Although there was no statistically significant difference between the groups (Pearson’s chi-square; p = 0.136), 14 students (93.4%) in group C agreed with the statement, whereas only 6 (46.2%) in group A and 8 (57.1%) in group B provided the same response.

The only item with a statistical difference between the groups in Test 4 (Pearson’s Chi-square; p < 0.001) was related to whether their stage of education would be suitable for teaching POCUS; 34 (80.9%) of the participants fully or partially agreed with the statement. When broken down by groups, all of the participants (n = 15) in group C fully agreed, 13 students (92.9%) in group B fully or partially agreed, and only 6 (46.2%) in group A agreed with the statement.

## DISCUSSION

The present study demonstrates that short-term teaching of POCUS and lung ultrasound can improve cognitive performance in these areas, regardless of the medical education level. The comparison between knowledge about the subject before the intervention showed a significant difference in general, except between students in the clinical cycle and the medical internship cycle. The post-course knowledge was only different between the extremes (i.e., between the basic cycle and the medical internship cycle). The growing median of the scores in Test 3 (i.e., execution and recognition of pathologies) showed a significant difference between the groups, suggesting that the more advanced the students are in medical education, the better the ability to perform and interpret exams correctly is, after a brief training. On the other hand, the medical education level does not seem to influence execution time.

Students generally agreed that POCUS is an important topic for medical practice, capable of improving diagnostic ability, and therefore important to be addressed in the medical curriculum. The perception that teaching time was sufficient was pointed out in general, with less homogeneity between the earlier stages. This, combined with the fact that most of the basic cycle group disagreed that their course phase is suitable for teaching POCUS, suggests that teaching the subject has a greater yield from the clinical cycle onward, more consistently in the internship cycle, which was perceived objectively by the results of the post-training cognitive test and the self-perception of knowledge acquisition by students in the qualitative questionnaire.

The present study is unprecedented, and, to our knowledge, there is no study in the Brazilian literature on medical education that compares the learning ability of POCUS in relation to medical education years. In addition, training was offered by professionals with extensive experience in POCUS and addressed cognitive aspects, performance skills, pattern recognition, and qualitative learning assessment.

The inclusion of ultrasound teaching in the curriculum, in the cognitive sphere and practical training, is positive by students’ perception.^14^ ^-^ ^16^ Although different ways of including teaching are described in the literature, there is emphasis and encouragement on the introduction of longitudinally teaching during medical education, from the basic cycle in correlation with anatomy to the clinical cycle.^10^ ^,^ ^17^ ^,^ ^18^

Even with a brief training, the teaching of POCUS involving the study of didactic material, theoretical classes, and practical training in a simulator or in live models can improve the quality of execution of the exam and cognitive knowledge when compared with no training.^8^ ^,^ ^19^

Regarding the best moment for teaching ultrasound use in academic life, training either medical students or resident physicians seems to provide them with adequate, specialist-like skills when undergoing adequate training and supervision.^8^ ^,^ ^20^ According to a study, performing cardiac ultrasound at the bedside by medical students after limited training improves the accuracy of cardiac diagnoses^21^ and can confer a sensitivity and specificity of diagnoses superior to those by cardiologists equipped only with a physical exam, evidencing the importance of cardiac imaging even if performed by future, non-specialist physicians with short training.^22^

Regarding the students’ perception about the appropriate moment for learning, the perception of students in the first and second years of medical school regarding the theoretical and practical teaching of POCUS showed that the initial phases were already considered to be adequate for teaching and that the training was already capable of increasing confidence in performing the exam.^23^

Although it is not a consensus, especially in Brazil, with the increased number of publications on the subject, the importance and need to introduce POCUS teaching is already evident in medical schools and not just in medical residency programs. Although the model, teaching methodology, and the stage that teaching should be introduced are still unclear, longitudinal programs with integration into the preclinical and clinical curriculum seem to be preferable.^10^ ^,^ ^11^

Among the numerous existing POCUS protocols, the BLUE protocol was chosen for its simple organization, which facilitates its reproducibility, ease of execution and interpretation of findings, and its ability to improve the accuracy of diagnoses in cases of acute respiratory failure.^12^

This study must be interpreted in the context of its limitations. A study with a small sample, in a single center, with more than one evaluator, the use of non-validated tests, and a short follow-up time are important limiting factors. In addition, the variables excluded from the study because the practical evaluation took place in a controlled environment with healthy models may not express the same result as care in a real medical emergency with patients in acute respiratory failure.

In Brazil, the teaching of POCUS is recent for specialist physicians, residents, and medical students. The absence of formal training on the subject, the small number of professionals trained to perform it and teach it, associated with the difficulties of large-scale access to ultrasound devices in medical schools, and the absence of a consensus on the expected basic skills are key obstacles to be overcome so that the introduction of this topic in medical schools can be achieved on a large scale.

For the future, gaps such as the existence or not of an ideal teaching moment and the creation of valid tools that are capable of quantifying theoretical and practical knowledge must be attained.

“A generation of doctors will need to be trained to see this technology as an extension of their senses, just as many generations saw the stethoscope. This development will require the medical education community to embrace and incorporate technology throughout the medical curriculum.”^9^

Since all technology requires training and experience, POCUS is not different. Its diffusion depends on the rupture of skepticism and traditionalism, as well as rewriting what are in fact the minimum skills expected of a doctor in training in 2024.

In conclusion, the findings of this study demonstrate that the theoretical teaching and practical training of lung ultrasound and the BLUE protocol, even for a short time, can significantly improve cognitive performance at all stages of the medical course, including the end of the basic cycle, the end of the clinical cycle, and during the medical internship. Moreover, practical training enhances the execution and interpretation of lung ultrasound and the BLUE protocol progressively throughout the medical course, particularly among students in the medical internship phase. These results align with the data indicating an increasing self-perception of learning lung ultrasound as students progress through their medical education.
